# Multigesture Electromyographic Control Complexity in Upper Limb Prostheses Actuated via Single Sensor Input Contraction Magnitude: Qualitative Study for Evaluating Performance and Cognitive Load

**DOI:** 10.2196/88528

**Published:** 2026-05-20

**Authors:** Abrianna Lalle, Samantha Migliore, Jeffrey Stevenson, Ethan Bell, Maanya Pradeep, Delaney Gunnell, Sophie Bennett, Viviana Rivera, Peter Smith, Matt Dombrowski, John Sparkman, Albert Manero II

**Affiliations:** 1 Limbitless Solutions University of Central Florida Orlando, FL United States

**Keywords:** prostheses, upper limb prosthesis, gamified training, usability, biomedical technology, artificial limbs, biomedical engineering, user centered design

## Abstract

**Background:**

Lack of functionality is one factor that contributes to prosthetic rejection rates. Electromyographic upper limb prostheses are controlled through muscle contractions in the user’s residual limb. The incorporation of multigesture controls into a novel, in-house developed upper limb prosthesis requires users to differentiate between the strengths of muscle contractions to trigger programmed gestures. Little research exists on the limitations of expanding device capabilities. This expansion may lead to a decline in accuracy and perceived usability or an increase in training time and cognitive workload.

**Objective:**

This study aimed to determine the feasibility of implementing multiple gestures when learning electromyographic controls during a single training session.

**Methods:**

Participants with full upper extremity control were fitted with a Flex Controller, a surface electromyography device that measures muscle contraction. Contractions were visualized as peaks and calibrated through an adjustable scale on a tablet. A training app was developed in-house to test novice users on an electromyography control system. Users interacted with 1, 3, or 5 zones on the screen. Each horizontal zone represented a threshold required to trigger a distinct gesture on the prosthesis. The cohorts were labeled A1 (n=9), A2 (n=10), A3 (n=9), and B1 (n=26). Every participant completed 3 trials per arm, and each trial consisted of 15 randomized cues. Each cue was represented by a green color change, with 1 point earned after a successful peak. Collected outcomes included performance, the System Usability Scale, and the National Aeronautics and Space Administration Task Load Index.

**Results:**

Scores decreased significantly as zones increased (Kruskal-Wallis H_3_=24.9, *P*<.001). The mean scores were 15.0 (SD 0.0) for 1 zone, 9.1 (SD 1.1) for 3 zones, and 5.5 (SD 1.1) for 5 zones. Perceived usability, measured by System Usability Scale, showed modest omnibus difference across cohorts (Kruskal-Wallis H_3_=5.22, *P*=.16); however, a pairwise comparison showed the 5-gesture cohort rated usability lower than the progressive cohort (2-tailed Welch t_11_=–2.19, *P*=.05). The 5-gesture cohort rated the system lowest (mean 63.3, SD 16.2). Cognitive workload, assessed through the National Aeronautics and Space Administration Task Load Index, increased with the number of gestures. The performance subscale showed a significant omnibus difference across cohorts (Kruskal-Wallis H_3_=21.4, *P*<.001). Mean performance subscale scores were 84.4 (SD 14.7) for the single-gesture condition, 30.6 (SD 21.7) for the 5-gesture condition, and 44.2 (SD 21.2) for the progressive cohort, reflecting increasing perceived difficulty with more gestures. The sample size for quantitative analysis was 54.

**Conclusions:**

These findings support the implementation of progressive training for 3 gestures. Usability perceptions were the highest among the more complicated progressive cohort, which is likely related to perceived improvement. Progressively learning 3 gestures enables a balance between device capability, user intention, perceived usability, and cognitive workload.

## Introduction

Upper limb prostheses must meet the functional demands of daily life [1,2]. Varied factors cited as contributors to prosthesis rejection demonstrate the need to further understand how to optimize training and device usage to meet these demands [3]. Electromyographic prostheses have fewer reported cases of device abandonment, attributed to increased capabilities [4]. Despite this, the lack of device functionality that contributes to prosthetic rejection rates [5] underscores the need to further expand device capabilities while maintaining user accuracy and perceived usability for novice users. The integration of advanced grips, finger configurations, and gesture recognition enables greater adaptability and reliability in a system [6]. Similarly, incorporating multiple gestures may improve prostheses’ functional capabilities, but they must be feasible for users to adopt the control scheme in daily life.

Electromyographic prostheses are controlled by muscle activation, which triggers the device to perform a preprogrammed gesture. Noninvasive surface electromyography (sEMG) detects microvoltages from the skeletal muscle, which are detectable from the skin’s surface [7,8]. To receive the electrical signals, sEMG can be embedded anywhere the device makes contact with the user’s residual limb. Multigesture electromyographic prostheses similarly require muscle activation but can perform multiple distinct movements. There are a variety of ways to process data collected by sEMG sensors to trigger a specific motion. Control schemes can use machine learning models in conjunction with several placed sEMG sensors [9], pattern recognition sequences [10], or magnitude-based discretization using a single sensor [11]. While a single-gesture prosthesis typically performs an open-close grasp with each muscle contraction, a multigesture prosthesis offers a variety of hand motions, such as pinch-point gestures, task-specific grasps, or individualistic expressions. Multigesture prostheses can be approached from a direct, single-sensor control perspective or from a pattern recognition standpoint [10]. Pattern classifiers have been found to potentially identify a user’s intention with up to 90% accuracy under ideal conditions, but present significant issues, including electrode shift, position variation, and force variation [11]. However, using a single electromyography (EMG) electrode with multigesture capabilities through force grading has been shown to be effective for pediatric populations [12].

This research aims to evaluate multigesture training with single sensor control. The research team has been developing a novel multigesture prosthesis that leverages a single sEMG sensor. Patients using the device can intentionally contract their muscles at different strength magnitudes to perform various gestures with the prosthetic limb. For example, a “light” contraction would prompt a different command than a “hard” contraction. Thresholds can be set to respond to a user’s individual strength range through calibration. This EMG signal processing includes amplification, rectification, band-pass filtration, and smoothing [13]. This control scheme is thought to perform better for people with congenital limb differences, who often have limited muscle volume and discretization compared with acquired amputations later in life.

All recorded EMG voltages from the sensors are processed and reported as values from 0 to 1023, with higher values indicating greater muscle contraction intensity. The input is filtered and rectified using bandpass filtering to reduce the reference scale to a comfortable range of contraction. When reading and processing muscle contraction for multigesture control, a single “flex” is quantified as the highest recorded EMG value within a series of recorded values above a set resting baseline. Each user can adjust this resting baseline to account for natural variance in base noise sensor readings. Any values below this resting threshold are effectively ignored by the prosthesis, allowing for intentional contractions to be easily differentiated from resting noise. When the continuous EMG signal from the prosthesis reaches a value above the resting threshold, all values are recorded within the current “flex” until the signal dips below the resting threshold again. Once the EMG signal falls below the resting threshold, the highest recorded value for the previous contraction is identified as the peak. This peak value is then compared with the preset multigesture thresholds to determine which threshold was hit. Each gesture threshold has a minimum contraction value, ranging from 0 to 1024, that can be manually set by each user. A threshold’s range spans from its minimum value to the next highest minimum value of the following threshold, or 1024 if there are no higher thresholds. When a discretized threshold is met, the corresponding hand gesture is triggered.

Implementation of multigesture EMG control using a single sensor is limited [11,14,15], but has leveraged pattern recognition and magnitude control. One example of this control implementation used to perform complex gestures is an assistive device that incorporates a hands-free wheelchair interface [14]. In this interface, engagement of the user’s temporalis muscle controls a wheelchair’s movement. Different levels of contraction correspond to wheelchair commands, including turning left, right, and moving forward similar to previous prosthesis controls [16]. This may be applicable in populations without the manual dexterity required to operate a traditional wheelchair joystick, such as those affected by a stroke or amyotrophic lateral sclerosis. This interface includes 3 levels of discretization, and research demonstrated that users could successfully gain control of the system [17], with three user control methods that allow independent use of the wheelchair and increase human-machine interaction and user autonomy. This successful schema demonstrates the potential for using a single-channel EMG system to control multiple outcomes.

Gamified training applies game design elements to nongame situations [18] and offers an engaging option to familiarize a patient with a system or motion. For electromyographic prostheses, gamified training can help teach novice users how to engage the targeted muscle and learn novel controls. There is a need to explore the impacts of serious gaming further, as discrepancies in the literature suggest that evaluating training platforms should be done on a case-by-case basis [19]. In a pediatric population, compliance with a training regimen is a multifactorial struggle that often falls on the caregiver [20]. Gamified training can encourage consistent preparation for an electromyographic prosthetic arm. The purpose of such training games is to expose and strengthen the children’s residual muscles before receiving their prostheses, which is intended to lower the time they need to become proficient with the device [21]. Game-based intervention shows an overall improvement in sEMG control and fine muscle activation [22].

The gamification in this study is built upon a preexisting gamified training strategy. The intention of the system is to prepare pediatric patients before using an EMG-powered prosthetic arm, with past research demonstrating gamified training leading to positive outcomes [23]. Training that starts at the time of initial device or before receiving a prosthesis may be beneficial [24,25]. Rehabilitation protocols are often progressive, requiring the establishment of baseline skills [26]. Some muscular rehabilitation settings have observed a benefit from using progressive training over static training [27]. In other settings, such as pain rehabilitation, no significant difference was found when comparing static and progressive training [28]. Various factors affect pediatric rehabilitation compliance, including engagement, motivation, and cognitive workload [29]. The cumulation of these factors has led to the need to examine progressive training in regard to pediatric electromyographic prosthesis use.

A gap in the current literature exists regarding the usability of electromyographic prosthesis systems, specifically those using a single-sensor system as opposed to the more common practice of including multiple sensors on the residual limb to gather EMG input [30]. The magnitude of contraction is less often the activation input for EMG devices; instead, multiple sensors working in conjunction using further signal analysis are more widespread. However, the incorporation of multiple sensors can add additional complexity to the system and make it more prone to error [15]. Although there is a range of electromyographic prostheses within the field of assistive technology, it has become apparent that standardization is necessary in the evaluation of EMG controls for single-sensor device models [31]. Additionally, opting to prioritize a single sensor may increase ease of learning in the pediatric population. While these multichannel systems using a field of muscle responses may be more beneficial toward amputees, who are more likely to have numerous feasible muscle groups for targeting with sEMG, this may differ in those with congenital limb differences. Amputees have been shown to learn new motor control schemes and muscle activation patterns via sEMG training [32].

As the etiology of congenital limb difference may vary, it is important to consider that the muscular architecture of each individual may not allow for multiple muscle targets. Methods of control for multigesture vary greatly, with each potentially benefiting from its own method of quantification. The use of a single-channel EMG system is uncommon, necessitating the development of a specific evaluation process for user acclimatization to the device. Additionally, an evaluation to determine the limit of the number of possible discretization thresholds should be developed. This testing software, in conjunction with the survey instruments, is intended to effectively measure the perceived difficulty level of the varying modes (1, 3, or 5 discretization zones within a single sensor) that are analogous to the multigesture electromyographic prosthetic device.

## Methods

### Study Design

**Figure 1:**
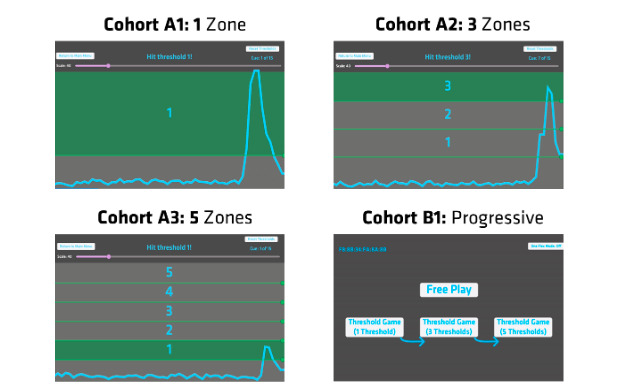
Representation of the progression scheme of each of the four cohorts. Cohort A1 included 1 section for participants to flex within. Cohort A2 consisted of the screen divided into three sections for participants to flex within. Cohort A3 consisted of the screen divided into five sections for participants to flex within. Cohort B1 had participants complete the first round with the screen divided into 1 section, the second round with 3 sections, and the third round with 5 sections.

To observe the results of novice users attempting to target various levels of contractions, a gamified training app, the Muscular Discretization Assessment Tool (MDAT), was developed in-house for user testing. The research team designed the MDAT to resemble the calibration system used by participants in a clinical trial testing the Limbitless Solutions prosthetic arm. Ensuring the effectiveness of the gamified MDAT controls before usage by prosthesis users was intended to optimize gameplay before distribution. When designing the gamified training app, complete parity with the functionality of the multigesture prosthesis was one of the main design aims. Each participant’s muscular contractions were displayed in a graph showing real-time voltage changes, as illustrated in Figure 1.

The app recorded and processed EMG input through the same mechanisms as the electromyographic prosthesis; once the user’s EMG signal passed the base resting threshold, all EMG values were recorded and saved until the signal fell below the resting threshold again. The EMG processing provides filtered data that directly corresponds to a muscular contraction, with a sampling rate of 1000 Hz. These steps include amplification, rectification, band-pass filtration ranging from 2 to 1000 Hz, and smoothing [13]. The highest recorded value between these 2 points was then identified and was considered the peak value of this contraction. This peak was used to determine which multigesture zone the contraction fell within. The app also allowed for the manual adjustment of each threshold, as well as a global scaling value, which amplified the measured EMG signal to allow users with weaker readings to still hit all thresholds easily. The prosthesis similarly allows for these levels to be adjusted and calibrated for a personalized experience.

The EMG Flex Controller sends data to the multigesture app approximately 60 times per second. These data are validated and processed by the app every time a new visual frame is calculated, which occurs at a capped rate of 30 times per second on the tablets available for user gameplay. This translates to a rate of processing and data reading from the controller once every 33.3 milliseconds. The app reads in muscle strength from the user directly as captured by the EMG sensors on the Flex Controller, and no additional adjustments are made to the readings by the game. The app also reflects the threshold system for triggering different gestures on the prosthetic, which provides one-to-one parity. Before app implementation in the study, internal testing was conducted. Since the software of the app was built on the framework of the calibration app used for the existing EMG-controlled prosthetic, the data are as reliable and seamless as they would be in real-world applications. The accuracy of this software has been tested before this study and has been implemented in ongoing clinical trials.

The calibration period consisted of participants completing a maximum muscular contraction. Based on where this signal ended, the sensitivity was adjusted. For example, if the participant was not able to easily reach the top of the screen via their muscular contraction, the signal sensitivity would be increased. This scale effectively “zooms in” the reading captured by the controller to bring the maximum captured value closer to the user’s readings without losing the accuracy and fidelity of data. This allowed for a standardized normalization process that was based on the user’s maximum contraction. The zone areas are initially segmented equally, but are customized to each participant such that they can feasibly hit all thresholds, with their baseline signal remaining below the lowest threshold. For additional support, the participant was prompted to hit each of the zones before actual gameplay.

The MDAT had a freeplay mode and various settings to replicate the multigesture thresholds analogous to the prosthesis control. The participant was fitted with an in-house developed Flex Controller around the forearm that contained surface electrodes to detect microvoltages from muscle contractions. The freeplay mode was used before data collection to calibrate the user’s Flex Controller to their personal muscle capabilities. The gameplay modes provided an environment for users to contract at various thresholds, recreating the muscle engagement required to operate the corresponding multigesture prosthetic device. In these gameplay modes, the screen was divided into 1, 3, or 5 sections, as presented in Figure 1.

The user interface (UI) for the gamified MDAT was designed to communicate the EMG signal and thresholds to the user as clearly as possible. Other elements, such as score, game instructions, and options, were placed at the top of the screen space to maximize the area available for EMG-related elements. Clarity was provided through explicit instructions for which randomized muscle strength grading thresholds are hit, delivered in a similar fashion as a game of *Simon Says* or *Bop It* (Hasbro Inc). A limited yet bright color palette was chosen for the UI to ensure that important elements stand out to users. A thick, bright blue line represented the user’s EMG signal from the Flex Controller, allowing users to understand how their muscle activity was translated in-game. Horizontal green lines across the screen indicated gesture thresholds. These were the only green elements in the UI and were intentionally chosen to make thresholds easy to identify at any moment during playtime. To further enhance threshold identification, each zone was visibly labeled as a numerical range. This aided users in identifying which threshold they were being prompted to hit during gameplay, as the game presented messages in the form of “Hit threshold X!.” X, in this instance, would correspond to the assigned threshold number that was cued to be hit. The cued threshold zone was highlighted in green, intuitively demonstrating to users which threshold they must hit without having to read the instruction prompt.

The participant had 1 attempt to reach within the threshold, and there was no time limit on completing an attempt. An attempt was considered successful if the participant’s peak contraction was within the cued highlighted threshold. Successful attempts were scored out of 15 cues per round to record overall performance.

A total of 54 participants completed data collection, with ages ranging from 18 to 30 years old. Anyone was invited to participate if they were between the ages of 18 and 64 years, able to consent, had full control of their upper extremities, had no injuries to their hands or arms that would impact their upper extremity control during data collection, and if they had never used a Limbitless Solutions myoelectric prosthesis or Flex Controller. Participants were assigned a cohort (A1, A2, A3, or B1) based on participation order. Cohort A1 included 1 section for the users to contract within, while sections A2 and A3 corresponded to 3 and 5 threshold sections, respectively. Cohort A1 had an analytical population of n=9. Cohort A2 had an analytical sample of n=10. Cohort A3 had an analytical sample of n=9. Cohort B1 had an analytical sample of n=26. Each of these cohorts completed 3 rounds with their respective number of zones. B1 was the progressive cohort, in which participants progressed through the 1, 3, and 5 zone modes in sequence. Users were first fitted with the Flex Controller on the right forearm. The controller was calibrated in freeplay mode based on the ease of comfortably reaching each threshold. This was guided by the researcher and manipulated by changing the sensitivity of the surface electrodes. Before gameplay, each user was informed of their respective cohort and shown each of the thresholded screens. For participants in the progressive cohort, researchers further explained the thresholds as fixed increments that changed progressively after each of the 15 cues was presented. Each participant played through the 15-cue round 3 times, taking 1-minute breaks in between each round to avoid fatigue. This procedure was then repeated with the Flex Controller on the left forearm.

After the testing period concluded, postassessment surveys were delivered to participants. These surveys included the Systems Usability Scale (SUS) and the National Aeronautics and Space Administration Task Load Index (NASA-TLX) [33,34]. The SUS is a 10-item scale commonly used as an objective measure of a digital interface’s usability [33]. The NASA-TLX measures cognitive workload through 6 categories [34]. The Edinburgh Handedness Inventory (EHI) was also completed to determine the participants’ dominant hand [35]. Finally, the user’s grip strength was measured using a handheld dynamometer. Grip strength was measured on both the right and left hands, and each was tested twice. The highest score for each hand was recorded. Participants also reported confidence with video games, confidence with exercise, confidence with visual cues, and activities they have participated in during the current week.

### Ethical Considerations

These procedures were approved by the University of Central Florida Institutional Review Board on October 1, 2024 (STUDY00007067). The inclusion criteria for participation consisted of people aged 18-64 years with full control of their upper extremities. Full informed consent was presented in both a visual and an auditory manner. Participants were encouraged to ask questions throughout the informed consent process and to request extra rest periods as needed. Participant confidentiality was preserved wherever possible by assigning a randomly generated 8-digit user ID based on participation order. These IDs were generated and stored on a premade linking spreadsheet. Once an ID was used, it was marked as “claimed” on the spreadsheet to ensure each was used only once. The Qualtrics forms containing all of the study data were linked to this ID, and no identifiable participant information was retained or used for analysis. The only potential participant compensation was extra credit from an instructor, which was offered at the discretion of the instructor. Each instructor who offered extra credit from this study was required to provide an alternative assignment of equal time and effort for the same amount of credit in the case that a participant could not complete the study.

### Statistical Analysis

Analysis was conducted in Python (version 3.12.9) using the libraries *pandas*, *NumPy*, and *scipy.stats*. The analytic sample was 54 participants (N=54). Tests were 2-tailed with α=.05; results were not adjusted for multiple comparisons. Normality was assessed for within-subject differences for paired comparisons using the Shapiro-Wilk test. When a paired comparison showed evidence of nonnormal differences (Shapiro *P*<.05), a Wilcoxon signed-rank test was run as a nonparametric sensitivity analysis and both results were reported. Nonparametric checks were used for ordinal or multilevel factors. SDs use *t*_(n−1)_=1 for effect size testing.

Score construction was in accordance with SUS standard guidelines (odd items: x–1; even items: 5–x; sum×2.5 to yield 0-100 scores) [33]. NASA-TLX raw inputs (1-21) were rescaled to 0-100 scores via (x–1)×5 [34]. The EHI laterality quotient was calculated via (R–L)/(R+L)×100, with R representing the responses associated with right-handedness, and L representing left-hand responses.

Within cohorts, trial scores were compared using paired, 2-sided *t* tests. The mean difference (95% CI), *t*_(_*_df_*_)_, *P*, and paired effect size Cohen *d* with Hedges *g* small-sample correction were calculated.

To assess the difference between a progressive and static mode of learning, in-between cohort comparisons were made between cohort B1’s trials and each of the respective A cohorts. For example, B1, B2, and B3 were compared with trial 1 of cohort A1, trial 2 of cohort A2, and trial 3 of cohort A3, respectively. This was done to make analogous comparisons based on the number of zones and trial numbers. Differing trials were isolated to account for users’ exposure time to the game and control system. Comparisons between cohorts were done using Welch 2-sided *t* tests due to unequal variances. The mean difference (95% CI), Welch *t*_(_*_df_*_)_ and *P*, and effect sizes (Cohen *d*, Hedges *g*, and average *d*_av_), were determined using pooled Cohen *d*, Hedges *g*, and average *d* (*d*_av_).

Associations between secondary variables (eg, confidence with video games, exercise, and cue use) and outcomes (trial scores, SUS, NASA-TLX, etc) were assessed using Spearman rank correlation computed within cohorts; ρ and *P* were reported. For ordinal factors with multiple levels, Kruskal-Wallis omnibus tests were conducted, with *H* and *P* reported where relevant. For multiselect variables (eg, videogame experience categories and activities), binary flags were created and compared groups via Welch *t* tests, reporting *d*_av_. Odds ratios were not calculated in this study, as they were not appropriate considering the data. Gender comparisons used Mann-Whitney *U* tests with rank-biserial r as effect size, as normality could not be assumed in the smaller subsample of men (n=12).

## Results

### Performance Scores

#### Overview

**Figure 2:**
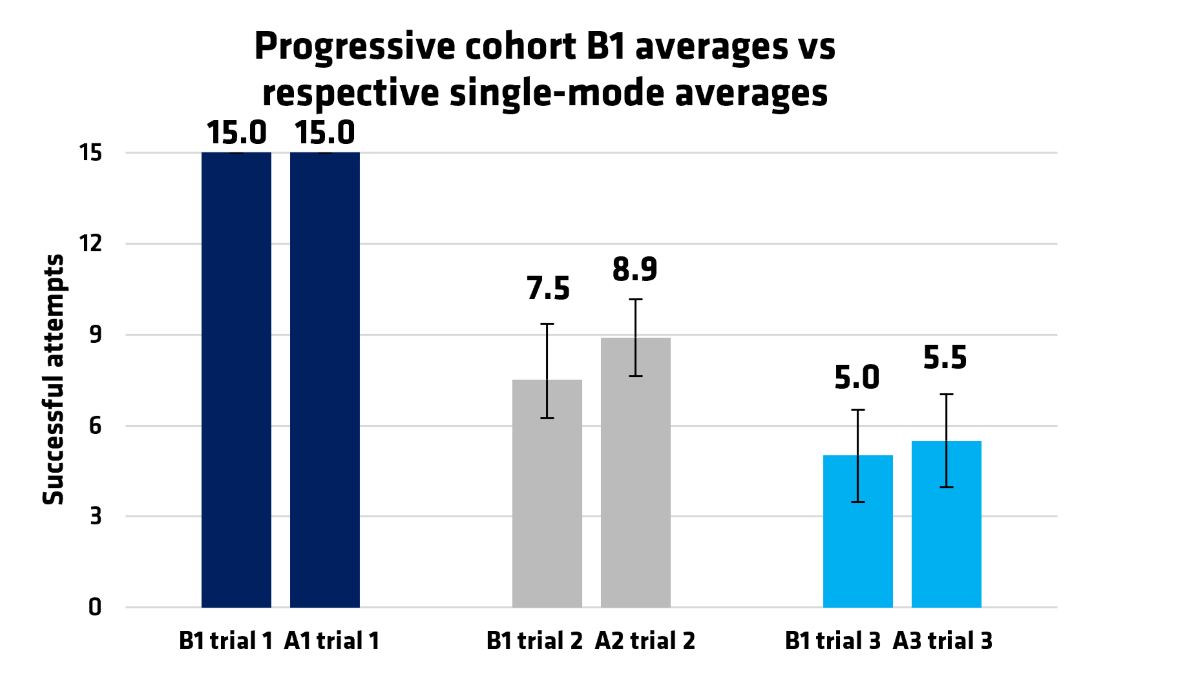
A comparison was made between the nonprogressive and progressive cohorts to determine performance differences. These performances are scored out of 15 potential successful attempts and visualized to include pairwise comparisons between Cohort B1’s Trial 1, Trial 2, and Trial 3, and the Al, A2, and A3 respective trials. Cohort A1 averaged a score of 15, A2 averaged 9.1, and A3 averaged 5.5. The progressive B1 cohort also followed most of these averages with average scores of 15, 7.5, and 5.0, respectively. Error bars correspond to plus/minus one standard deviation. A general trend observed was that performance decreased as the number of zones increased in both cohorts; however, there were no statistically significant differences between the cohorts.

Comparisons were made between cohort B1’s trial 1, trial 2, and trial 3, and the Al, A2, and A3 respective cohorts. These respective trials are matched for comparison based on the number of thresholds, as well as the time spent exposed to the game, which was measured through sequential trials. The outcomes demonstrate that user accuracy decreased as the number of zones increased. When playing with 1 threshold, as illustrated in Figure 2, as B1 trial 1 and A1 trial 1, the average score was 15 (SD 0) successful attempts out of 15 possible points per round. Scores from playing with 3 thresholds are displayed in the middle column, labeled B1 trial 2 and A2 trial 2. Scores from playing with 5 thresholds are displayed on the right side of the graph, labeled as B1 trial 3 and A3 trial 3. This trend is displayed in Figure 2.

Cohort A1 averaged a score of 15 (SD 0.0), A2 averaged 9.1 (SD 1.1), and A3 averaged 5.5 (SD 1.1). The progressive B1 cohort also followed most of these averages with average scores of 15 (SD 0.1), 7.5 (SD 1.8), and 5.0 (SD 1.5) for each of the 3 trials, respectively. SD was included in parentheses, as seen in Table 1. Table 2 compares the B1 progressive gameplay mode to each of the respective A cohorts, paired by the number of zones. Cohort A1 averaged a score of 15 (SD 0.0), A2 averaged 8.9 (SD 1.5), and A3 averaged 5.5 (SD 1.1); the progressive B1 cohort also followed most of these averages. Overall, no statistically significant differences were found between the B1 cohorts and their corresponding A cohorts, indicating that the groups are comparable.

**Table 1:** Performance scoring by successful attempts.

Cohort	Trial 1, mean (SD)	Trial 2, mean (SD)	Trial 3, mean (SD)	Average score, mean (SD)
A1	15.0 (0.0)	15.0 (0.0)	15.0 (0.0)	15.0 (0.0)
A2	8.9 (1.5)	8.9 (1.3)	9.6 (1.8)	9.1 (1.1)
A3	5.6 (1.9)	5.3 (0.7)	5.5 (1.5)	5.5 (1.1)
B1	15 (0.1)	7.5 (1.8)	5.0 (1.5)	9.2 .9)

**Table 2:** Between-cohort comparisons.

Cohort and trial	Mean difference (95% CI)	Welch *t* (*df*)	Hedges *g*	Cohen *d*_(__*av*__)_	*P* value
B1, A1, Trial 1	–0.04 (–0.09 to 0.02)	–1.44 (25.0)	–0.32	–0.40	.16
B1, A2, Trial 2	–1.38 (–2.49 to –0.27)	–2.57 (23.8)	–0.79	–0.88	.02
B1, A3, Trial 3	–0.48 (–1.75 to 0.79)	–0.81 (13.7)	–0.31	–0.32	.43

#### Performance Scoring by Successful Attempts

Performance was determined by the number of correct randomized cues a participant hits with a peak contraction out of 15 total cues. Performance scores were compared across cohorts and separated by trial, with SDs included (Table 1). Cohort A1 averaged a score of 15 (SD 0.0), A2 averaged 9.1 (SD 1.1), and A3 averaged 5.5 (SD 1.1). The progressive B1 cohort also followed most of these averages, with an overall average of 9.2 (SD 0.9).

Within-cohort paired comparisons across trials did not show statistically significant changes in performance. In cohort A2, trial 1 vs trial 3 differences deviated from normality (Shapiro *P*=.01); therefore, a Wilcoxon signed-rank test was conducted as a sensitivity analysis. Results were consistent with the paired *t* test (*t*_9_=−1.07, *P*=.32; Wilcoxon W=13.5, *P*=.59).

#### Comparison Between Cohorts

Between-cohort comparisons were also assessed to determine statistical significance in user performance. Cohort A1 averaged a score of 15 (SD 0.0), A2 averaged 8.9 (SD 1.5), and A3 averaged 5.5 (SD 1.1). The progressive B1 cohort also followed most of these averages. There were no statistically significant differences between the B1 and the respective counterparts for A1 (*P*=.16) and A3 (*P*=.43). Cohort A2 performed significantly higher in Trial 2 than Cohort B1 (*P*=.02, ρ=0.8). A negative mean difference favored the A Cohorts. Hedges *g* was also listed in the table (Table 2). The average (SD) standardized mean difference was *d*_(_*_av_*_)_.

### SUS

Cohorts A1, A2, and B1 had the highest perceived usability as measured through the SUS, as seen in Table 3 [33]. The SUS is measured using a 100-point scale, with 100 portraying the best imaginable product, and 0 indicating an unusable product [33]. Cohorts A1 and A2 both had an average SUS score of 75 (cohort A1: mean 75.0, SD 18.9 and cohort A2: mean 75.0, SD 16.8). Cohort A3 had the lowest SUS score with an average of 63.3 (SD 16.2). Cohort B1 had the highest average SUS score with 76.2 (SD 11.7). The overall group SUS score was 73.6. A Welch *t* test showed a significant difference between cohorts A3 and B1 (*P*=.05).

**Table 3:** Overall System Usability Scale scores by cohort.

Cohort	Overall SUS^a^, mean (SD)
A1	75.0 (18.9)
A2	75.0 (16.8)
A3	63.3 (16.2)
B1	76.2 (11.7)
Overall	73.6 (15.1)

^a^SUS: System Usability Scores.

SUS is a measure of perceived usability and was taken as a postassessment survey [33]. Average overall SUS scores and the respective SDs are separated by cohort. Cohorts A1, A2, A3, and B1 had overall average scores of 75.0 (SD 18.9), 75.0 (SD 16.8), 63.3 (SD 16.2), and 76.2 (SD 11.7), respectively. The overall average score between all the cohorts was 73.6 (SD 15.1), which falls in the “Good” category on the corresponding subjective scoring of the SUS [33].

### NASA-TLX and Subscales

**Figure 3:**
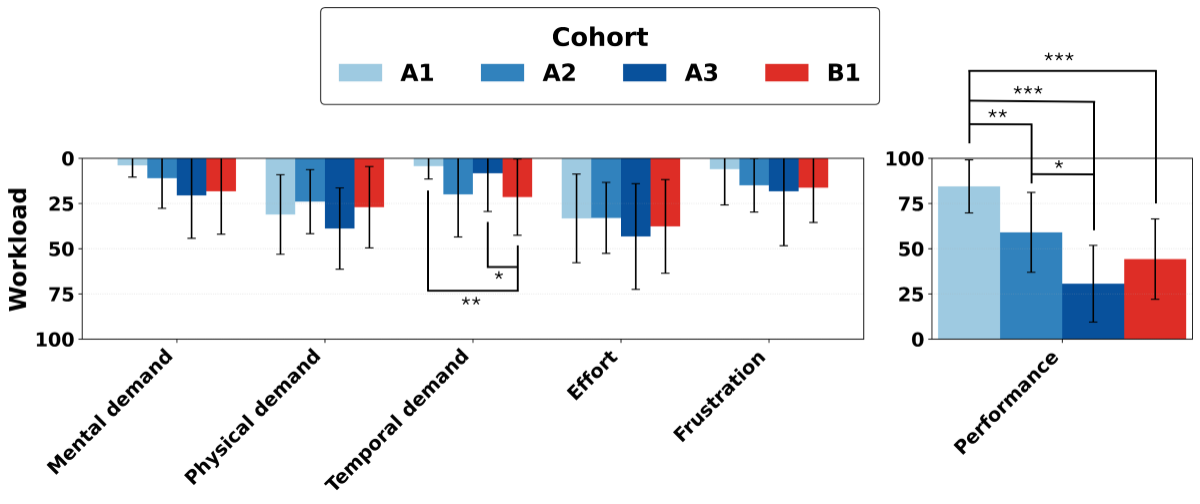
The National Aeronautics and Space Administration Task Load Index (NASA-TLX) measures perceived cognitive workload [34]. It is divided into 6 subscales, each on a 100 point scale where 0 is the desired low cognitive workload [34]. Performance is on an inverse scale, where 100 would indicate a more desirable performance [34]. This subscale is depicted on the right in its own graph to account for this difference. The trends overall confirmed the hypothesis that demand would increase, while performance would decrease, due to increased zones of control. The progressive training Cohort B1 countered the expectations, having less perceived difficulty than Cohort A3. Note: *:*P*<.05; **:*P*<.01; and ***:*P*<.001.

Inversely, NASA-TLX scores are on a 100-point scale, with 0 indicating the desired lowest cognitive workload, and 100 indicating the highest cognitive workload [34]. The NASA-TLX consists of 6 different subscales that are the main contributors to cognitive workload—mental demands, physical demands, temporal demands, frustrations, effort, and performance [34]. These subscales were selected due to underlying theories of workload that correspond to demands that a user perceives during a task [34]. These subscales are depicted visually per cohort in Figure 3. The performance subscale has an inverse relationship, where higher self-reported performance scores indicate a lower cognitive workload and are considered more favorable [34]. Average NASA-TLX scores were highest among cohort A3 in 5 out of 6 subcategories. From a Kruskal-Wallis test, each subscale had a *P* value. Only the performance subscale passed the significance threshold. These results are presented in Table 4. This follows the expected trend of cognitive workload increasing as the number of zones expands. Differences in perceived effort and perceived frustration were not statistically significant between cohorts. To clarify how these workload patterns varied between specific cohorts, pairwise comparisons of performance and temporal demand subscales are presented in Tables 5 and 6. The largest and most significant differences emerged in the performance subscale, where the single-gesture cohort A1 outperformed all other cohorts with large mean differences. The temporal demand subscale had significant comparisons involving cohort B1, with higher perceived time pressure than cohort A1 and cohort A3. Within the mental demand subscale, cohort A1 tested significantly lower than cohort B1 (*P*=.01, *d*_(_*_av_*_)_ =–0.80). In the physical demand subscale, no tests achieved statistical significance, but cohort A2 had a lower score than A3 (*P*=.15, *d*_(_*_av_*_)_=0.70).

**Table 4:** Average NASA-Task Load Index subscores (separated by cohorts, with corresponding SDs included for each of the scores).

Cohort^a^	Mental demand	Physical demand	Temporal demand	Performance	Effort perceived	Frustration perceived	Overall
A1, mean (SD)	3.9 (6.6)	31.1 (22.0)	4.4 (7.1)	84.4 (14.7)	33.3 (24.5)	6.1 (19.7)	16.4 (10.9)
A2, mean (SD)	11.0 (16.7)	24.0 (17.7)	20.0 (23.6)	59.0 (22.1)	33.0 (19.6)	15.0 (14.8)	24.5 (14.1)
A3, mean (SD)	20.6 (29.7)	38.9 (22.5)	8.3 (8.9)	30.6 (21.2)	43.3 (29.2)	18.3 (30.1)	33.3 (19.6)
B1, mean (SD)	18.3 (23.7)	27.1 (22.5)	21.5 (21.1)	44.2 (21.2)	37.7 (25.9)	16.3 (19.2)	29.7 (14.5)
*P* value	.49	.38	.09	<.001	.86	.29	.12

^a^Scores for each subsection are on a 100-point scale, with 0 indicating a desired low cognitive workload [34]. The performance subscale is the only exception to this, where 100 would indicate a more desirable score [34]. The NASA-Task Load Index measures perceived cognitive workload through a postassessment survey [34]. A general trend of cognitive workload increasing with a higher number of zones was observed.

**Table 5:** Pairwise comparisons for the NASA-TLX performance subscale. This subscale is scored inversely, with higher values indicating better perceived performance and lower cognitive workload [34]. This subscale had the largest and most consistent differences between cohorts. Cohort A1 rated its performance significantly higher than Cohorts A2, A3, and B1 with large standardized mean differences. Rows are organized with the most significant results at the top.

Comparison	Cohen *d*_(__*av*__)_	Cohen *d*_(__*av*__)_ (SE)	*P* value
A1, A3	2.91	0.70	<.001
A1, B1	2.20	0.47	<.001
A1, A2	1.36	0.52	.009
A2, A3	1.23	0.51	.01
A2, B1	0.68	0.38	.09
A3, B1	–0.64	0.39	.12

**Table 6:** Pairwise comparisons for the NASA-TLX temporal demand subscale [34]. Temporal demand showed fewer significant results than the performance subscale, but still reflected meaningful differences between cohorts. Cohort B1 reported significantly higher temporal demand than cohort A1 and cohort A3. Other pairwise contrasts within this subscale did not reach significance, although cohort A1 and cohort A2 showed a medium effect size trend. Rows are organized with the most significant results at the top.

Comparison	Cohen *d*_(__*av*__)_	Cohen *d*_(__*av*__)_ (SE)	*P* value
A1, B1	–1.06	0.41	.001
A3, B1	–0.79	0.40	.02
A1, A2	–0.89	0.48	.07
A2, A3	0.65	0.47	.17
A1, A3	–0.48	0.48	.32
A2, B1	–0.05	0.37	.89

### Performance and Composite SUS and NASA-TLX Scores Compared Between Cohorts

**Figure 4:**
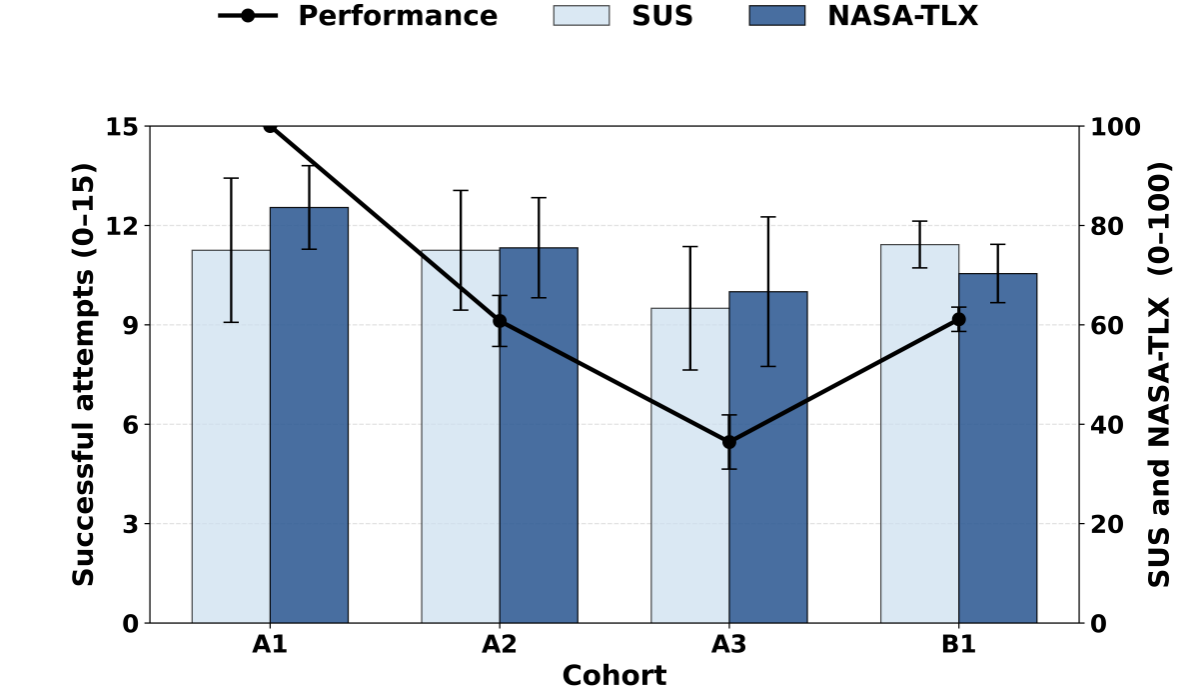
NASA-TLX (National Aeronautics and Space Administration Task Load Index) and SUS (System Usability Scale) composite scores are displayed in a combined bar chart, and successful attempts are overlaid as a line graph for Cohorts A1, A2, A3, and B1 [33-34]. This allows for the visualization of trends and tradeoffs between number of zones and cognitive load, usability, and accuracy. The number of successful attempts decreased as the number of zones increased from Cohort A1 to A3. No meaningful conclusions could be drawn from composite NASA-TLX scores between cohorts. The SUS composite scores did not differ significantly between Cohorts A1, A2, and A3, but a significant difference was found between Cohorts A3 and B1. NASA-TLX composite scores are inverted so that a higher score is favorable for the sake of graph continuity and tradeoff visualization.

Successful attempts at scoring, SUS scores, and NASA-TLX scores are compared across cohorts in Figure 4. This allows for the visualization of trends and tradeoffs between the number of zones and accuracy, usability, and cognitive workload.

### Dominant Hand Grip Strength

Grip strength was measured in kilograms using a dynamometer after the completion of the postassessment surveys. The average grip strength in kilograms is segmented by gender. The frequency of each reported gender and the SDs are included in Table 7.

**Table 7:** Dominant hand grip strength by self-reported gender.

Gender	Frequency, n	Grip strength (kg), mean (SD)
Men	12	44.0 (11.2)
Women	40	27.3 (4.6)
Other	2	21.3 (0.6)

### Secondary Assessments

Secondary measurements included the EHI and participants’ confidence with video games, exercise, and following visual cues. Participants were also asked to share activities they completed within the last 7 days that required the use of fine or gross motor skills. Based on EHI calculations, all participants tested into pure right-handedness, with a laterality total of >40. Across correlation tests per cohort, most associations were small and nonsignificant (median |ρ|=0.12, IQR 0.05-0.34). Only 2 showed significance after inspection, that is, exercise frequency vs grip strength (ρ=0.53-0.75, *P*=.03-.002) and cue-use confidence vs workload (ρ=0.45, *P*=.01).

Preassessment surveying also included demographics, video game experience, exercise, crafts, instruments, and other fine-motor tasks. Handedness inventory, self-reported dominant hand, and grip strength were assessed. There was no correlation between gender and performance (ρ=–0.11, *P*=.43), despite large grip strength differences between male and female participants (ρ=–0.57, *P*<.001).

## Discussion

### Principal Findings

This study aimed to evaluate potential trade-offs associated with increasing gesture capability in an electromyographic prosthetic arm actuated by a single sensor. Increased training time, increased perception of cognitive workload, decreased accuracy, and decreased usability may all be factors discouraging the implementation of multiple gestures. This study explored 1, 3, and 5 distinct gestures triggered by magnitude control and their training feasibility in a single-session rapid learning environment. In addition, differences between static and progressive learning modes were also evaluated. The progressive training mode (cohort B1) demonstrated the feasibility of reducing demand on the user.

Extrapolating from past work and the results of this study, a long-term training regime using a gamified interface that closely corresponds to the prosthetic device’s interface may allow users to improve multigesture use. Developing control of electromyographic inputs in a low-stress, low-consequence manner is critical to unlocking the prosthesis’s advanced functionality without patient rejection. Devices with more features and more advanced controls may result in frustration for patients and lead to rejection. Providing a “challenge mode” to test controls that can unlock additional device functionality may be a solution to this issue. The results of this work indicate that a 3-tiered gesture control scheme is capable with a limited training timeline. However, it is important to note that able-bodied users may experience different fatigue patterns or motivation levels compared with individuals with limb differences. In the prosthesis clinical trial protocol, the training games are delivered before the prosthetic arm in an effort to train basic muscle control and discretization. Once the prosthesis is delivered, it has a single threshold control for open and close functionality. Gamified training continues until a 3-threshold differentiation is easily achievable by the user, and only then is multigesture support unlocked in the prosthetic device. The goal is to have the majority of failures occur in the training games and increase initial success with the prosthetic device. The outcomes of this work show that a tiered approach is appropriate for training 3 thresholds. Future work will help determine if long-term training can support a 5-threshold model.

### Cohort Performance Scoring

To determine if including multiple gestures is feasible, it is necessary to consider performance, usability, and cognitive workload. The 3-gesture mode demonstrated similar usability scores as the single-gesture cohort, with better cognitive workload and performance scores than the 5-gesture mode. This suggests that incorporating 3 gestures may strike a functional balance between difficulty and usability for first-time interactions with the system. Additionally, a progressive mode of learning demonstrated the highest perceived usability, despite incorporating up to 5 gestures, which had the lowest scores and the highest cognitive workload in the static mode of learning. Capping initial progressive training at 3 gestures has the potential to increase perceived usability further while retaining the higher accuracy and lower cognitive workload of cohort A2.

Previous studies have shown that increased time on task is associated with higher user performance outcomes with the electromyographic controls [23,36]. Additionally, close correspondence of a training period with posttest tasks resulted in greater performance scores, demonstrating the importance of task-specific training.

### SUS

The overall SUS scoring of 73.6 falls into the “ok” designation, demonstrating that this system was likely deemed usable by the participants [33]. Individual cohort scoring indicated that cohort A3 had the lowest perceived usability. Since this cohort had also reported the greatest perceived mental workload and the lowest performance among participants, this was expected to have the lowest usability scoring. Cohort B1 has the highest perceived usability, which is supported by lower NASA-TLX scores and similar performance scores to the nonprogressive cohorts [34]. The 12.9-point SUS difference between cohort A3 and cohort B1 is both statistically significant (*P*=.05) and meaningful with a Cohen *d* of −0.91. This indicates a substantial difference between cohort averages relative to within-group variability. Clinically, a significant difference with a large effect size implies that users in a progressive learning environment experience are likely related to higher levels of usability than users in a static 5-zone learning environment.

### NASA-TLX

The NASA-TLX, measuring perceived cognitive workload, demonstrated that cognitive workload increased as the number of gestures increased [34]. Similar to the trend in scoring, difficulty tended to increase with the inclusion of more gestures, so it was expected that cognitive workload would follow the same pattern. NASA-TLX scores were aligned with scoring deficits instead of perceived usability as measured through SUS [33,34]. Cohort A3 demonstrated the highest cognitive workload in 5 of the 6 NASA-TLX subcategories [34].

### Limitations

One limitation of this study was the unintentional inclusion of exclusively right-handed participants. Although a small proportion of participants self-reported as left-handed (n=6), the EHI score indicated right-hand bias [35]. This may not be representative of true population proportions, however, and instead may be due to the relatively small sample size of this study.

Similarly, 40 of the 54 participants in the study were female. This can be explained by smaller sample sizes and convenient population sampling of college students. Throughout the data collection process, minor collection discrepancies were accounted for. One participant withdrew from the study due to a personal situation during data collection. The participant revoked their consent to participate, and the research session was terminated immediately. Their data were removed from the dataset. During another session, one user ID was claimed by 2 distinct participants during the data collection process. Since both participants completed the study independently of each other, the datasets were differentiated with an (a) and (b) at the end of their identifier, and both datasets were used in the final analysis.

The aim of this study was to explore trends in novice users as they were exposed to different controls. This study used a proxy population of college-aged students who had full control of their upper extremities, which is not fully representative of our target population of prosthesis users. Although these findings cannot be completely generalized to prosthesis users, the intention of this initial testing is to ensure positive outcomes among users before implementation in limb-different population groups. Additionally, the target population is primarily pediatric patients, which is seen as a vulnerable population group for testing purposes. In order to limit confounding variables, such as different levels of limb difference and the effect of differing anatomical muscle structure, a proxy, an able-bodied population, was then used. Past work in prosthesis development has shown a significant difference in muscular activation between amputees and nonamputees [32]. As such, the use of a proxy population then allows for standardization that may prove more difficult in the intended population, as well as identification of potential errors, streamlining eventual delivery. Serious games have also been tested in a proxy population of college-aged students and have been further implemented with patients with a limb difference [23,36]. The observed performance measures, including accuracy scoring, SUS, and NASA-TLX, have been used independently among studies that include different limb users [33,34]. Additionally, future work may strengthen findings through the use of a pre-post comparison. Past work in the facility has evaluated the efficacy of motor learning through serious games and evaluated participant scoring before and after intervention [23]. Future work may evaluate long-term changes in scoring using the training app. This study demonstrated the efficacy of the MDAT within a proxy population, with the intention that future work will include testing in the intended clinical population of upper limb prosthesis users.

The unequal and relatively small sample sizes in cohorts A1, A2, and A3 limit statistical power, particularly for between-cohort comparisons with the progressive cohort B1. Although effect sizes were calculated and in several cases were small to moderate, the absence of statistically significant differences between cohorts may reflect limited power rather than true equivalence. Therefore, findings comparing progressive and static modes should be interpreted cautiously and considered hypothesis-generating. Further testing with the application will be ongoing with the current proxy population and a representative limb-different population.

Another limitation of this study is the single-gesture scoring metric for cohort A1. Cohort A1 had a mean score of 15.0 (SD 0.0). This is because all contractions that are above threshold are considered a success, as there is only 1 zone. This creates a ceiling effect that may have affected variance and statistical analysis. This also highlights the functionality of single-gesture mode testing for novice users. There is a possibility of a fatigue effect due to 3 repetitive trials, which the researchers aimed to minimize by implementing mandatory 1-minute rest periods per round and offering continued rest if the participant wanted more recovery time.

### Conclusions

The most clinically relevant and statistically significant results were found from the SUS scoring [33]. Specifically, the significant increase in perceived usability for the progressive cohort seen in this study may influence user adherence to training, addressing one of the primary concerns surrounding prosthetic device rejection. There were no statistically significant differences between cohorts in scoring for progressive mode and static mode, and there were only 2 statistically significant subscales of the NASA-TLX [34]. Both the SUS scores and the NASA-TLX scores displayed a trend that difficulty increases with the inclusion of more gestures as anticipated [33,34]. This is supported by the results from cohort A3, which had the lowest accuracy scoring and the highest cognitive workload.

Especially among young prosthesis users, maintaining adherence to a training regime is particularly difficult [20]. The usability of training systems is necessary to encourage motivation in pediatric training regimes [37]. Overall, these results suggest that implementing up to 3 gestures with a progressive training mode may have the potential to balance the increasing complexities required for additional gestures. This work aims to increase the functionality while not introducing an undue burden. Since reducing the complexity of a training regimen may discourage device rejection, this is an essential consideration. This may enable expanding device capabilities while maintaining accuracy, perceived usability, and a lower cognitive workload.

This control schema will be implemented in a gamified training format to teach users the musculature engagement and control required to operate an upper limb prosthesis with multiple gesture states. This progression will be introduced to the intended population through long-term clinical trials of individuals with a limb difference who are training to use a prosthetic device.

Implementing these training controls into a gamified regimen may increase time on task [23]. Prolonging the time engaged in a training measure can increase its effectiveness; this engagement may decrease the time needed to learn controls, as reinforcement learning can begin with the first introduction to controls. Reinforcement learning in gamified training may significantly improve performance scores [38-41]. Applying this principle to future training applications has the potential to encourage pediatric compliance with a training program, as time on task and reinforcement learning lead to improved performance and engagement.

Future work aims to evaluate these controls in a longer-term research trial incorporating a 2-gesture step toward training up to 3 gestures progressively. This can be implemented into the pre-existing gamified training protocol used in current pediatric clinical trials. Increasing the in-game difficulty may prepare users for the increased difficulty associated with additional gestures on the prosthetic device. One eventual aim of future work is to discern what form of gamified training would be most effective to establish device use on the first day of delivery.

## Data Availability

The datasets analyzed during this study are available from the corresponding author upon reasonable request.

## Abbreviations

EHI: Edinburgh Handedness Inventory
EMG: electromyography
MDAT: Muscular Discretization Assessment Tool
NASA-TLX: National Aeronautics and Space Administration Task Load Index
sEMG: surface electromyography
SUS: System Usability Scale
UI: user interface
